# Optic Nerve Injury, Vitreous Hemorrhage, and Rhegmatogenous Retinal Detachment Following Long Needle Peribulbar Anesthesia: A Case Report and Review of the Literature

**DOI:** 10.7759/cureus.31329

**Published:** 2022-11-10

**Authors:** Rawan K Alrajhi, Ashwaq M Almalki, Abdullah S Alqahtani

**Affiliations:** 1 College of Medicine, King Saud bin Abdulaziz University for Health Sciences, Jeddah, SAU; 2 College of Medicine, King Abdullah International Medical Research Center, Jeddah, SAU; 3 Ophthalmology, Ministry of National Guard Health Affairs, King Abdulaziz Medical City, Jeddah, SAU; 4 Ophthalmology, King Abdullah International Medical Research Center, Jeddah, SAU; 5 Ophthalmology, King Saud bin Abdulaziz University for Health Sciences, Jeddah, SAU

**Keywords:** ocular perforation, vitreous hemorrhage, retinal detachment, peribulbar anesthesia, cataract surgery

## Abstract

Rhegmatogenous retinal detachment (RRD) following retrobulbar or peribulbar anesthetic injection is a rare but serious complication that often results in poor visual outcomes. Thus, extreme caution should be exercised while administering local ocular anesthesia due to the potential complications arising from local orbital anesthesia. These complications may occur locally or systemically and may arise immediately or be delayed. This case report is on a female patient who sustained optic nerve injury and RRD due to a peribulbar block administered in the setting of cataract extraction and, subsequently, experienced retinal detachment and vitreous hemorrhage at another hospital before being referred to our hospital. The retina was repaired with pars plana vitrectomy, 360 endolaser of the peripheral retina and around tears, and gas injection, achieving stable visual outcomes.

## Introduction

Retrobulbar and peribulbar anesthesia (PBA) are ophthalmic techniques that are commonly performed to induce anesthesia [1]. These techniques are straightforward, easy, and safe to perform by trained ocular surgeons or anesthetists [1]. PBA is considered safer and easier to administer than intraconal retrobulbar anesthesia [2]. However, although PBA is a relatively safe technique, it is not impervious to risks [2]. Some rare intraocular complications do result from mechanical injury of the needle, such as retinal breaks, hemorrhage around retinal breaks, vitreous hemorrhage (VH), retinal detachment (RD), or subretinal hemorrhage (SRH) [3]. Rhegmatogenous retinal detachment (RRD) following retrobulbar or peribulbar anesthetic injection is a rare but serious complication that often results in poor visual outcomes [4]. This case report is on a female patient who experienced RRD due to a peribulbar block administered before cataract surgery at another hospital before being referred to our hospital for surgical repair.

## Case presentation

**Figure 1:**
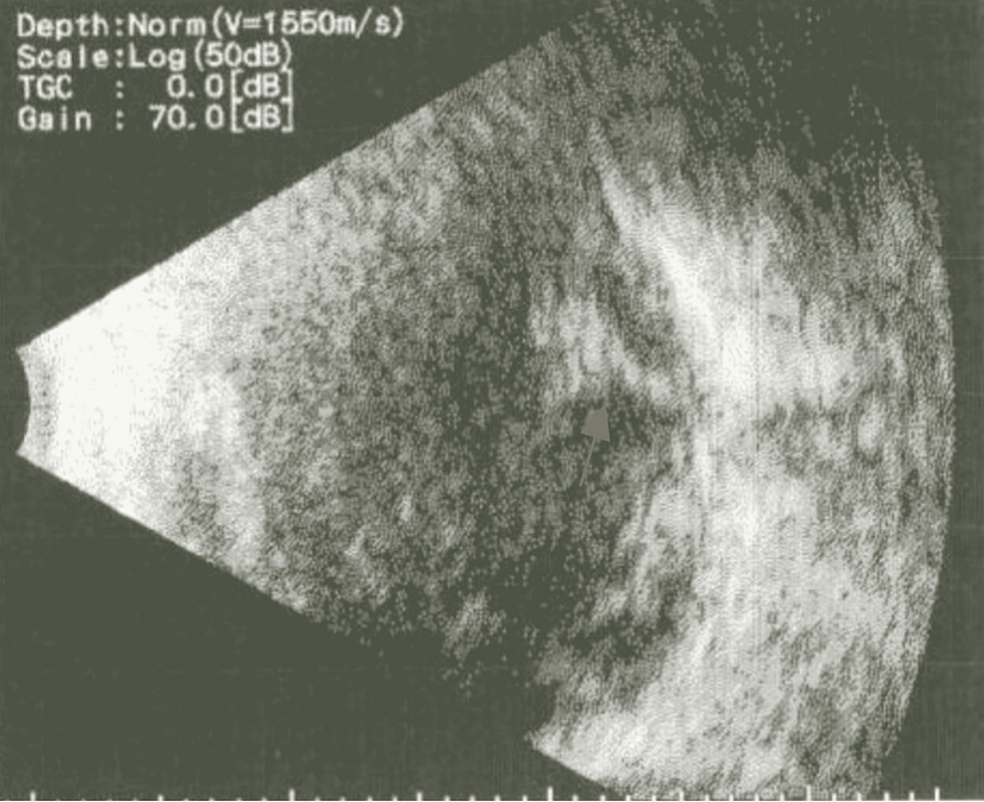
Orbital B-scan of the left eye.

A 59-year-old female with a left senile cataract and pre-operative visual acuity (VA) of 6/60 underwent left phacoemulsification cataract extraction surgery with intraocular lens (IOL) implantation at another hospital. PBA was administered using a standard technique with an injection of lidocaine hydrochloride (xylocaine) 2% in the left eye. The patient made an accidental sudden movement of the head during injection and immediately developed a syncopal attack that was managed by the anesthesia team till she became stable and alert, after which she agreed to the surgery. After the surgery was completed, the patient was stable and satisfied and discharged on the same day. On the first post-operative day, the VA was light perception. B-scan ultrasonography showed left VH with RD (Figure 1).

**Figure 2:**
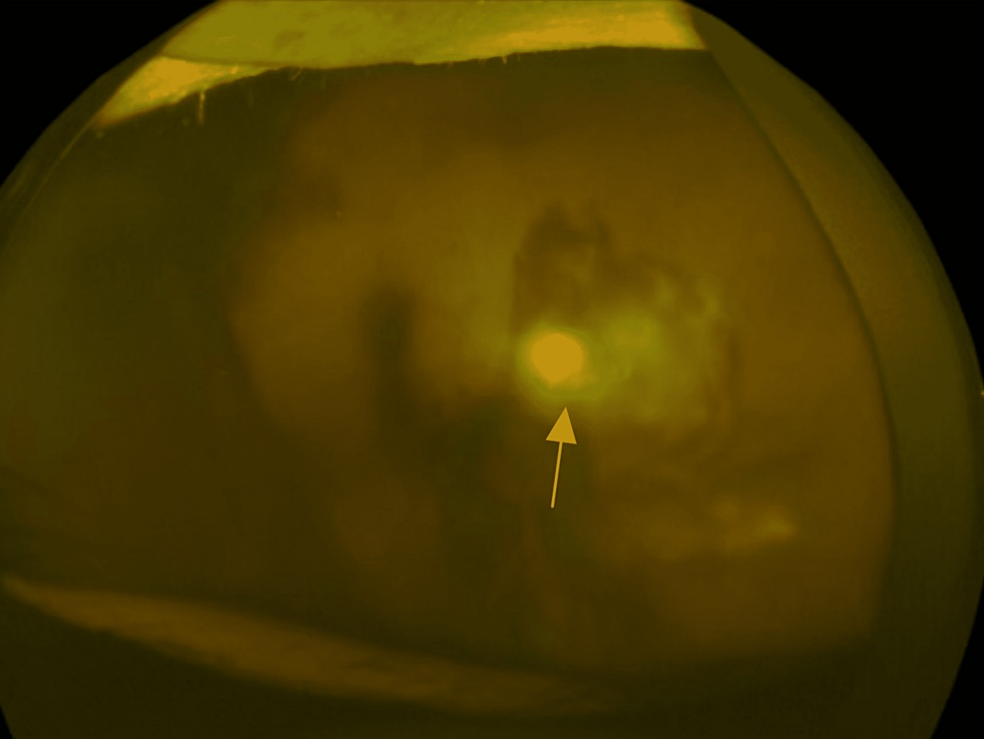
Fundoscopy before retinal repair. Left vitreous hemorrhage obscuring the site of perforation.

Fundus examination showed left VH obscuring the site of perforation (Figure 2).

Due to transfer management, the patient was referred to our center for post-cataract surgery and, subsequently, underwent 23-gauge pars plana vitrectomy along with a 15% C3F8 gas injection and 360 endolaser under local anesthesia. Following the surgery, the retina was attached, and the vitreous was clear.

At the one-month follow-up, the uncorrected distance visual acuity (UDVA) was found to be 6/6 in the right eye and light perception in the left eye. An anterior segment exam was within the normal limit and showed a clear cornea. Fundus examination showed a pale optic disc with peripapillary atrophy and the retina in place.

**Figure 3:**
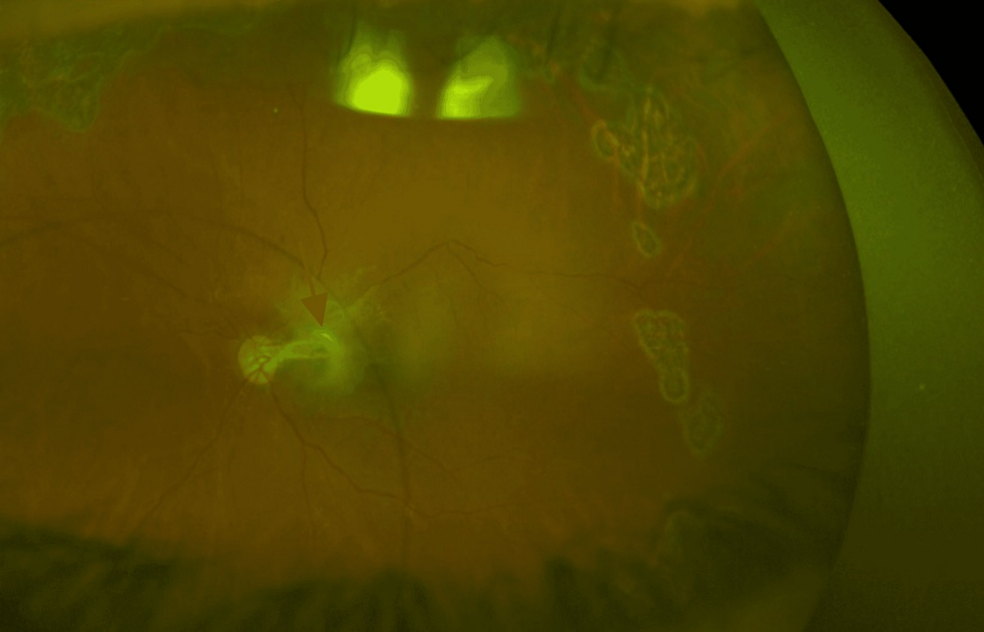
Fundoscopy following retinal repair. Pale nerve with a scar nasally to the macular area suggesting the location of the posterior pole perforation at the time of the anesthesia with optic nerve damage.

At the two-month follow-up, the UDVA was found to be 6/6 in the right eye and light perception in the left eye, and the intraocular pressure (IOP) was normal. Fundus examination showed a pale optic disc with peripapillary atrophy, a fibrovascular membrane extending from the optic disc inferiorly, and a flat retina (Figure 3).

At the three-month follow-up, the UDVA was found to be 6/6 in the right eye and light perception in the left eye. The IOP was normal. The pupils were normal and reactive to light with no relative afferent pupillary defects (RAPD). An anterior segment exam was within the normal limit and showed a clear cornea. The anterior chamber (AC) was deep and quiet, with a normal iris in both eyes. Fundus examination showed a pale optic disc and the retina fixed in place.

Six months later, the VA of the left eye was stable. The UDVA was 6/12 in the right eye and light perception in the left eye. Fundus examination showed peripapillary fibrosis and optic atrophy. Consent to publish this article was obtained. 

## Discussion

PBA is considered a safe alternative to retrobulbar anesthesia for cataract surgery, as it involves the administration of short needles (15-25 mm) parallel to the roof and floor of the orbit outside the muscle cone, avoiding damage to periocular structures and minimizing intraocular complications [1]. Ocular perforation is a rare but one of the most dangerous complications of intraocular anesthesia. It has been reported in the literature that the incidence of accidental ocular perforation after retrobulbar anesthesia or PBA is 0.006-0.13%, with an increased risk for retrobulbar anesthesia compared to PBA, and its prevalence ranges between 1 in 1,000 and 1 in 10,000 cases [1]. Accidental ocular perforation can be caused by many risk factors including long axial length, posterior staphyloma, the use of inappropriate instruments, uncooperative patients, and inexperienced personnel administering the block. Ocular perforation can lead to many serious complications, such as retinal break, VH, SRH, and RRD [2].

It is common practice to use a 25 mm needle or longer to administer peribulbar blocks [4]. The possibility of inadvertently placing such a needle tip into the retrobulbar space has been indicated in previous research [5]. This unintentional retrobulbar injection can lead to rare but serious side effects and devastating complications [6]. Wrong material usage is an important consideration when performing local anesthesia. Using a one-inch 23-gauge needle instead of a 1.5-inch peribulbar needle for such a block technique will help prevent severe complications such as optic nerve injury and vision loss.

However, identifying globe perforation at the time of injection is not always easy. Wearne et al. [1] reported that globe perforation was noticed in 18 of 20 eyes within one week of surgery [1]. A total of 15 eyes with globe perforation were identified on the first post-operative day due to dense VH [1]. We reported a case where globe perforation presented on the first day after cataract surgery, the VA was light perception, and B-scan ultrasonography showed left VH with RD.

The common causes of poor vision in our case include needle injury to the macula or maculopapular bundle, optic atrophy, and macular SRH [7]. Management options were determined by the nature and extent of intraocular complications. The presence of VH with or without RRD indicated the need for immediate vitrectomy due to the aggressive development of PVR changes. In our report, the patient was referred following cataract surgery to our hospital, where she underwent a 23-gauge pars plana vitrectomy along with a 15% C3F8 gas injection and 360 endolaser. However, longer follow-up periods might be needed to check for recurrence.

## Conclusions

Retrobulbar and peribulbar blocks are safe and effective adjuncts when administered by a qualified ocular surgeon or anesthetist. Safety and caution must be maintained while administering local ocular anesthesia. However, in high-risk populations, the ophthalmologist may use less intrusive techniques for blockages, such as sub-Tenon’s block or topical anesthesia. The risk of complications can be reduced by following technical guidelines and using proper-sized needles.
